# Identification and validation of a chronic kidney disease prediction model after radical nephrectomy: a multicenter cohort retrospective study

**DOI:** 10.1080/07853890.2026.2737504

**Published:** 2026-09-29

**Authors:** Xinning Wang, Tianwei Zhang, Jingxian Wang, Yongchao Yan, Haoyuan Wang, Kefan Song, Yongbo Yu, Zhilei Zhang, Benkui Zou, Changlei Yao, Jianbo Zheng, Jie Liu, Hongli Liu, Xuefeng Zhang, Chen Guan, Lingyu Xu, Ningxin Zhang, Wei Jiao

**Affiliations:** a Department of Urology, The Affiliated Hospital of Qingdao University, Qingdao, China; b Department of Urology, Graduate School of Medicine, Juntendo University, Tokyo, Japan; c Department of Urology, The Second Hospital of Tianjin Medical University, Tianjin, China; d Department of Urology, Qilu Hospital of Shandong University, Jinan, China; e Department of Urology, Weifang People’s Hospital, Weifang, China; f Department of Urology, Cancer Hospital of Shandong First Medical University, Jinan, China; g Department of Urology, People’s Hospital of Rizhao, Rizhao, China; h Department of Urology, Zibo Central Hospital, Zibo, China; i Department of Urology, Linyi People’s Hospital, Linyi, China; j School of Clinical Medicine, Shandong Second Medical University, Weifang, China; k Department of Urology, Weihai Central Hospital, Weihai, China; l Department of Nephrology, The Affiliated Hospital of Qingdao University, Qingdao, China

**Keywords:** Renal cell carcinoma, radical nephrectomy, chronic kidney disease, machine learning, multicenter study, retrospective study

## Abstract

**Introduction:**

Long-term renal function is an important follow-up after radical nephrectomy for renal carcinoma. Predictive models can help identify patients at risk for chronic kidney disease (CKD) progression and enable early intervention.

**Methods:**

We retrospectively analyzed 649 patients who underwent radical nephrectomy at eight medical centers. The primary cohort from the Affiliated Hospital of Qingdao University comprised 329 patients, randomly divided into training sets (*n* = 229) and internal validation sets (*n* = 100) at a 7:3 ratio. An additional 320 patients from seven other centers constituted a multicenter external evaluation cohort. Five machine learning models were developed, including Logistic Regression (LR), Support Vector Machine (SVM), Random Forest (RF), Extreme Gradient Boosting (XGBoost), and Light Gradient Boosting Machine (LightGBM). Model performance was evaluated using the area under the receiver operating characteristic curve (AUC), confusion matrices, and calibration curves.

**Results:**

224 (34.51%) patients experienced postoperative CKD stage progression within three years. LightGBM achieved the highest discriminative performance among the five evaluated algorithms, with an AUC of 0.7508 (95% CI, 0.6399–0.8617) and an accuracy of 0.7200 in the internal validation set, and an AUC of 0.7549 (95% CI, 0.7022–0.8076) and an accuracy of 0.6813 in the external evaluation set. SHAP analysis identified preoperative eGFR, tumor size, post-to-preoperative serum creatinine ratio, preoperative serum creatinine, and age as the five most influential predictors.

**Conclusions:**

We developed and externally evaluated machine-learning models for predicting CKD stage progression after radical nephrectomy. Long-term decline in renal function is an important complication that requires urologists’ attention and early prevention during follow-up.

## 1. Introduction

Radical nephrectomy is the standard surgical approach for treating renal carcinoma [1]. However, this surgery leads to the loss of a large number of effective and good nephrons, which may pose a risk of renal dysfunction or even chronic kidney disease (CKD) for patients [2]. Radical nephrectomy brings a high probability of AKI and CKD risks to patients [3,4]. Currently, the quality of life of tumor patients after surgery has become a focus of attention. Therefore, to avoid long-term renal dysfunction and reduce the risk of future end-stage renal disease or dialysis, a predictive model for postoperative renal function changes needs to be established for patients who underwent radical nephrectomy. In this study, based on previous research [5], we included data from multiple medical centers for validation, hoping to use this model to screen out high-risk patients with renal dysfunction at the first treatment and implement more strict medical preventive measures for such patients after surgery.

## 2. Methods

### 2.1. Inclusion and exclusion criteria

All included patients had renal cell carcinoma and underwent radical nephrectomy from 2012 to 2020. The exclusion criteria were as follows: (1) age < 18 years, (2) follow-up time < three years; (3) preoperative CKD stage greater than 3, (4) no renal function follow-up data within 36–39 months after surgery, and (5) lack of preoperative renal function(including serum creatinine and eGFR). According to the CKD staging criteria proposed by the Kidney Disease Outcome Quality Initiative (KDOQI) of the National Kidney Foundation in 2002, the outcome of this study was whether CKD staging was upgraded.

### 2.2. Data sources

This was a multi-center retrospective study. This retrospective report includes no identifiable patient information. The study was approved by the Ethics Committee of the Affiliated Hospital of Qingdao University, and the requirement for individual patient informed consent was waived. Finally after applying the exclusion criteria, we retrospectively analyzed the electronic medical records of 649 patients from the Affiliated Hospital of Qingdao University (*n* = 329), Qilu Hospital of Shandong University (*n* = 119), Weifang People’s Hospital (*n* = 70), Cancer Hospital of Shandong First Medical University (*n* = 45), People’s Hospital of Rizhao (*n* = 27), Zibo Central Hospital (*n* = 27), Linyi People’s Hospital (*n* = 20), and Weihai Central Hospital (*n* = 12). The patient selection process is shown in Figure 1.

**Figure 1.**
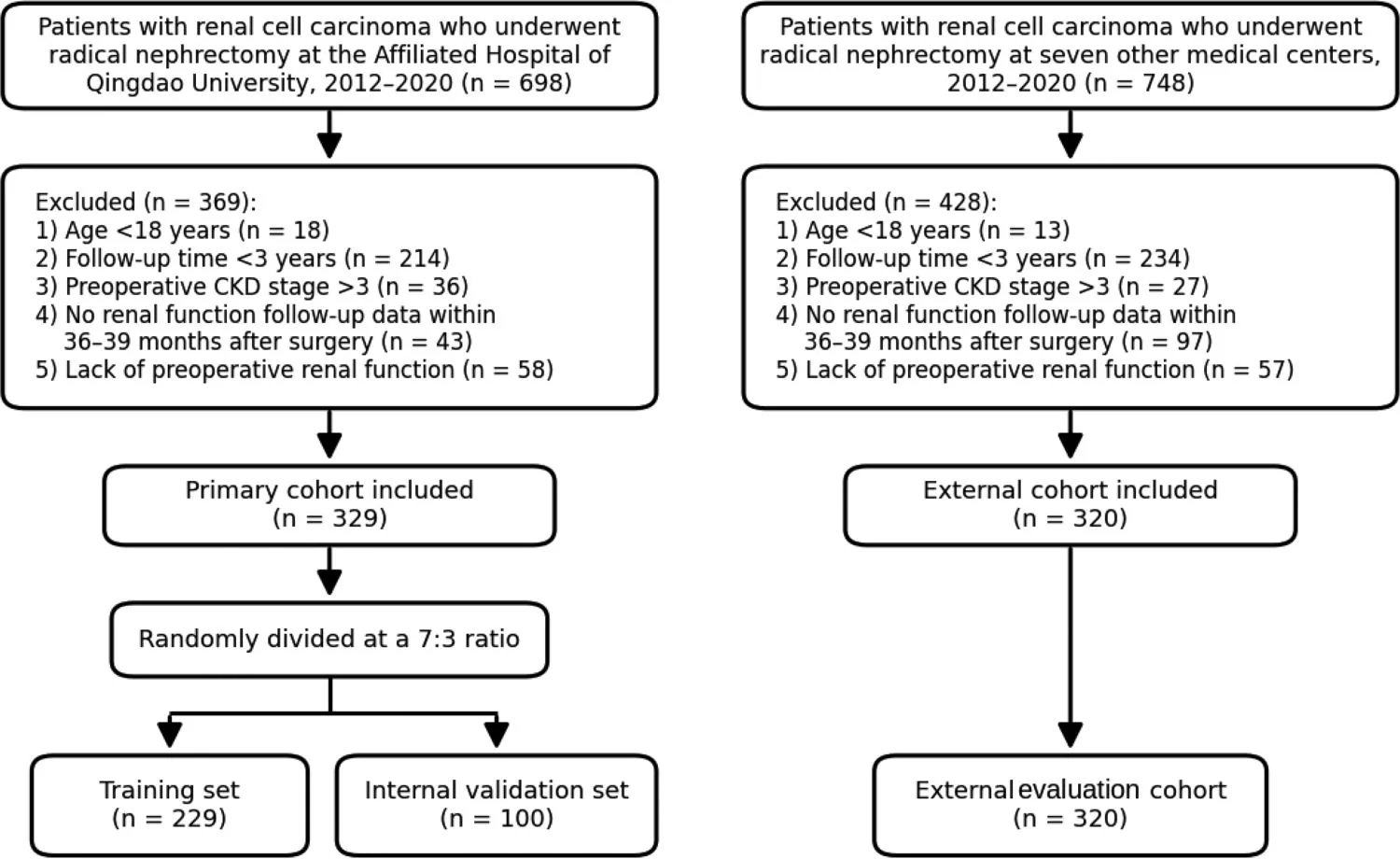
Flow chart of patient selection. Between 2012 and 2020, 698 patients undergoing radical nephrectomy for renal cell carcinoma at the Affiliated Hospital of Qingdao University and 748 patients at seven other medical centers were screened. After applying the exclusion criteria, 329 patients were included in the primary cohort and randomly divided into a training set (*n* = 229) and an internal validation set (*n* = 100) at a 7:3 ratio, and 320 patients from the seven external centers constituted the external evaluation cohort.

During the study period, 698 patients undergoing radical nephrectomy for renal cell carcinoma at the Affiliated Hospital of Qingdao University and 748 patients treated at the seven external centers were initially screened. In the primary cohort, 369 patients were excluded: age <18 years (*n* = 18), follow-up time <3 years (*n* = 214), preoperative CKD stage >3 (*n* = 36), no renal function follow-up data within 36–39 months after surgery (*n* = 43), and lack of preoperative renal function (*n* = 58), leaving 329 patients for model development and internal validation. In the external cohort, 428 patients were excluded for the same reasons (*n* = 13, 234, 27, 97, and 57, respectively), leaving 320 patients for external evaluation. The final analysis dataset constituted a complete-case analysis. After the exclusion of other ineligible patients, 58 and 57 patients in the primary and external cohorts, respectively, lacked preoperative renal function measurements (i.e. preoperative serum creatinine, from which the preoperative eGFR was derived) and were excluded before cohort allocation; all other candidate predictors were complete for every screened patient, and no imputation of missing data was performed. Failure of outcome ascertainment (absence of renal function follow-up data within 36–39 months after surgery; *n* = 43 and *n* = 97, respectively) was handled as a separate exclusion criterion. All 649 patients included in the final analysis had complete data for all candidate predictors and the outcome.

### 2.3. Data extraction

In this study, we included the following factors based on our recent research [5]: (1) demographic information (age, gender, and BMI); (2) comorbidity information (hypertension, diabetes); (3) laboratory indicators (preoperative serum creatinine, post-to-preoperative serum creatinine ratio and preoperative eGFR); and (4) medical history information (surgical method and tumor size). The data from the Affiliated Hospital of Qingdao University was defined as primary cohort, and then randomly be divided into two subsets: the training set (*n* = 229) and internal validation set (*n* = 100) in a 7:3 ratio. The cohort from other hospitals (*n* = 320) served as external evaluation set.

The eGFR was estimated using the CKD-EPI 2021 equation, which is recommended by the KDIGO clinical practice guideline for estimating eGFR (including serum creatinine, age, and gender). The criteria for renal function assessment were as follows: (1) baseline renal function was determined from the most recent preoperative renal function measurement; (2) postoperative follow-up renal function was determined from measurements obtained 36–39 months after surgery, and when multiple measurements were available within this period, the one closest to 36 months was selected.

### 2.4. Predictor selection

The candidate predictors used in the present study were prespecified based on our previously published single-center study, in which clinical factors associated with long-term renal function after radical nephrectomy were investigated. Predictor selection was therefore not performed using a new data-driven feature-selection procedure in the current cohort. The following ten variables were included in all machine-learning models: age, gender, BMI, hypertension, diabetes, preoperative serum creatinine, preoperative estimated glomerular filtration rate, post-to-preoperative serum creatinine ratio, tumor size, and surgical approach. These variables were selected because of their previously demonstrated predictive relevance, clinical accessibility, and consistent availability across the participating centers. No predictors were selected or excluded according to univariable P values in the current study.

### 2.5. Data preprocessing and model development

Prior to model development, continuous predictors were standardized using Z-score transformation. The means and standard deviations used for standardization were estimated exclusively from the training set and subsequently applied without modification to the internal and external evaluation sets. To address class imbalance, the synthetic minority oversampling technique (SMOTE) was applied exclusively to the training set, whereas the internal and external evaluation cohorts retained their original class distributions.

To develop models for predicting CKD stage progression within three years after radical nephrectomy, five ML algorithms were applied: Support Vector Machine (SVM), Random Forest (RF), Logistic Regression (LR), eXtreme Gradient Boosting (XGBoost), and Light Gradient Boosting Machine (LightGBM). Hyperparameters for each algorithm, where applicable, were optimized using a grid-search strategy combined with five-fold cross-validation over predefined parameter spaces. The hyperparameter configuration yielding the highest mean cross-validated area under the receiver operating characteristic curve (AUC) was selected as optimal and subsequently used to refit each model using the entire training set. The predefined hyperparameter search spaces and final optimal values are presented in Supplementary Table S1. The models were evaluated without further modification in the internal validation set and the external evaluation set. The distribution of the datasets and the model construction workflow are shown in Figure 2.

**Figure 2.**
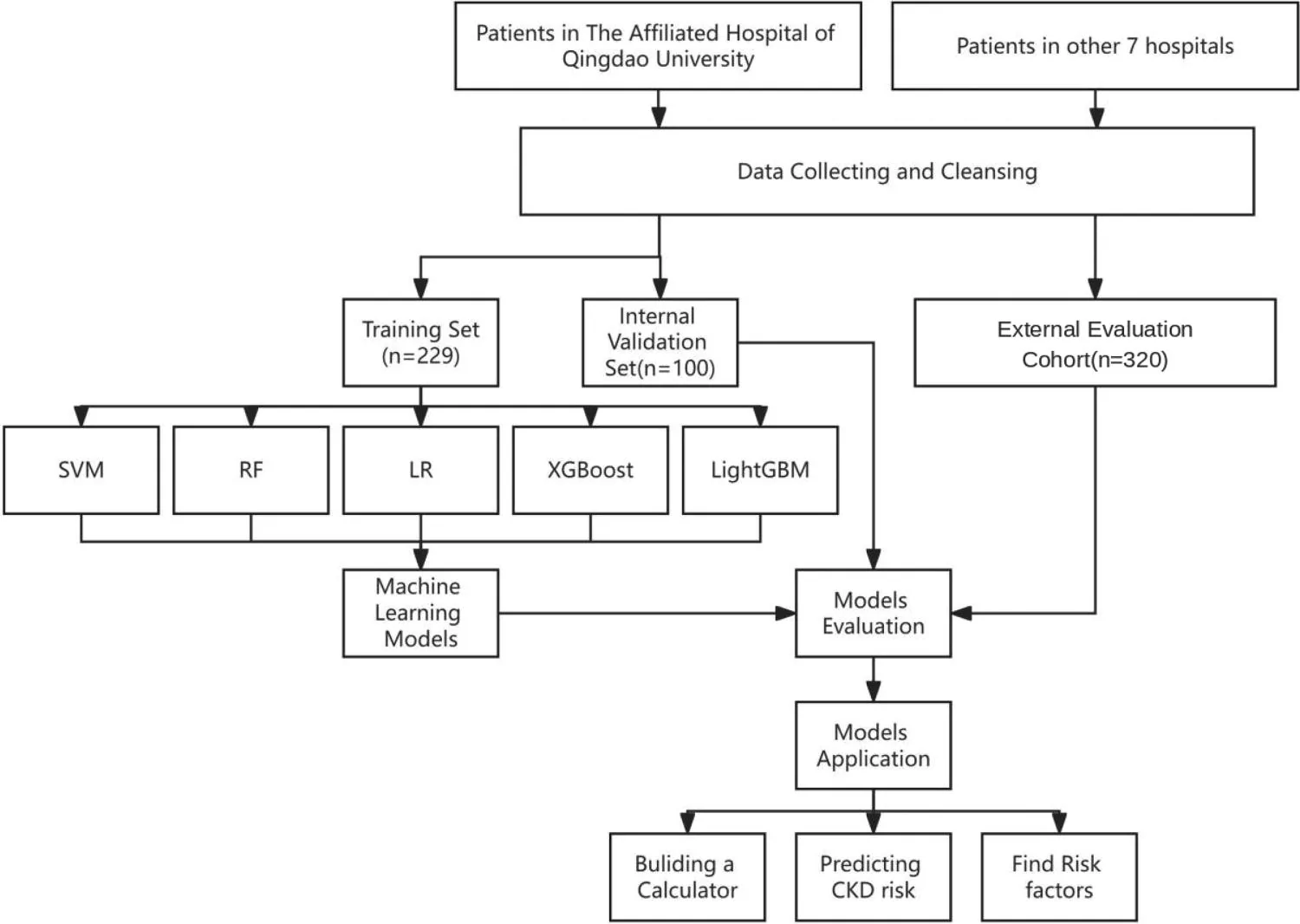
Flow chart of model construction and verification.

Model discrimination was assessed using the AUC and its 95% confidence interval, accuracy, sensitivity, and specificity. The optimal classification threshold was determined by maximizing the Youden index. Calibration performance was assessed visually using calibration curves and quantitatively using the Brier score, with lower values indicating smaller overall prediction errors between predicted probabilities and observed outcomes. Clinical utility was evaluated using decision curve analysis, which estimated the net benefit of each model across a range of threshold probabilities.

The resulting candidate models were first assessed in the internal validation set for out-of-sample model comparison. All candidate models were subsequently evaluated in the multicenter external cohort to examine their performance and transportability across participating centers. Because performance in the external cohort was also considered when identifying the preferred algorithm, this cohort is referred to as an external evaluation cohort. After considering discrimination, calibration, probabilistic prediction accuracy, clinical utility, and interpretability across these analyses, LightGBM was identified as the preferred model for subsequent interpretation.

### 2.6. Calibration assessment

Model calibration was assessed visually using smoothed calibration curves. Overall probabilistic predictive performance was further evaluated using the Brier score, with lower Brier scores indicating better agreement between predicted probabilities and observed outcomes. In addition, the calibration intercept (calibration-in-the-large) and calibration slope were quantified for the internal and external evaluation cohorts. The calibration intercept was estimated from a logistic regression of the observed outcome with the logit of the predicted probabilities included as an offset term, and the calibration slope was estimated from a logistic regression of the observed outcome on the logit of the predicted probabilities. An intercept of 0 and a slope of 1 indicate perfect calibration. The 95% confidence intervals (CIs) were derived from the regression standard errors. Calibration curves, Brier scores, calibration intercepts, and calibration slopes were evaluated for both the internal and external evaluation cohorts.

### 2.7. Model interpretation

To address concerns regarding the black-box nature of ML algorithms, we employed SHAP values to enhance the interpretability of our models. SHAP, including KernelSHAP and TreeSHAP, provided a novel approach for explaining and visualizing the predicted outcomes of various ML models. TreeSHAP was a fast and precise algorithm designed to efficiently compute SHAP values for individual decision trees and tree-based ensemble models. We utilized a SHAP summary plot to visualize both the global feature importance, ranked by the mean absolute SHAP value across all patients, and the directionality of each feature’s impact on predicting the CKD stage progression in patients with renal cancer after radical nephrectomy.

### 2.8. Statistical analysis

Continuous variables with an approximately normal distribution are presented as the mean ± standard deviation and were compared using the independent-samples t test. Non-normally distributed continuous variables are expressed as medians and interquartile ranges and compared using the Mann-Whitney U test. Categorical variables are presented as numbers and proportions and compared using the chi-square test or Fisher’s exact test. All machine learning analyses were conducted in R (version 4.4.2) using the tidymodels, bonsai, mxjqkit, mxjqcls2, and shapviz packages. The predictive power was evaluated using the AUC, with the optimal cut-off value determined by maximizing the Youden index (sensitivity + specificity − 1). Pairwise comparisons of the AUCs among the machine-learning models were performed using the paired DeLong test in the internal and external evaluation cohorts. All tests were two-sided, and a *p* value < 0.05 was considered statistically significant.

## 3. Results

### 3.1. Patient characteristics

A total of 649 patients who underwent radical nephrectomy from 8 medical centers were included into this study, 224 (34.51%) experienced an upgrade in CKD stage 3-year post-surgery. Detailed information regarding the selected patients is provided in Table 1. As shown in Table 2, the primary cohort and the external cohort differed significantly in age, preoperative serum creatinine, preoperative eGFR, the post-to-preoperative serum creatinine ratio, gender, surgical approach, and the study outcome, whereas no significant differences were observed in the remaining characteristics. These discrepancies may be related to differences in the patient populations enrolled at the participating centers. The training and internal validation sets were comparable across all baseline characteristics except age and hypertension, which differed significantly (Table 3).

**Table 1.** The baseline of patients with or without upgrading of CKD.

	Primary cohort			External cohort		
Characteristics	No upgrading of CKD	Upgrading of CKD	*p* value	No upgrading of CKD	Upgrading of CKD	*p* value
*n*	251	78		174	146	
Age	57.82 ± 11.06	60.09 ± 9.82	0.105	53.92 ± 12.42	59.77 ± 8.69	< 0.001
BMI	25.26 ± 3.52	25.18 ± 3.32	0.946	25.40 ± 3.46	25.54 ± 3.31	0.718
Preoperative creatinine	84.0 (69.5, 97.0)	78.5 (71.0, 90.0)	0.483	67.0 (55.0, 85.0)	65.65 (57.25, 75.75)	0.298
Preoperative eGFR	82.20 (71.00, 98.96)	92.20 (73.03, 98.31)	0.192	97.58 (80.42, 110.19)	97.63 (92.92, 102.76)	0.969
Post-to-preoperative serum creatinine ratio	1.28 (1.09, 1.46)	1.28 (1.17, 1.60)	0.065	1.28 (1.06, 1.50)	1.54 (1.31, 1.67)	< 0.001
Tumor size	5.0 (3.5, 7.0)	5.0 (3.5, 6.0)	0.209	5.0 (3.5, 7.4)	4.8 (4.0, 6.0)	0.804
Hypertension, *n* (%)			0.293			0.483
No	152 (60.6%)	42 (53.8%)		108 (62.1%)	85 (58.2%)	
Yes	99 (39.4%)	36 (46.2%)		66 (37.9%)	61 (41.8%)	
Diabetes, *n* (%)			0.016			0.932
No	216 (86.1%)	58 (74.4%)		152 (87.4%)	128 (87.7%)	
Yes	35 (13.9%)	20 (25.6%)		22 (12.6%)	18 (12.3%)	
Gender, *n* (%)			0.283			0.006
Female	157 (62.5%)	54 (69.2%)		76 (43.7%)	42 (28.8%)	
Male	94 (37.5%)	24 (30.8%)		98 (56.3%)	104 (71.2%)	
Surgical approach, *n* (%)			0.732			0.758
Open	44 (17.5%)	15 (19.2%)		16 (9.2%)	12 (8.2%)	
Laparoscopy or Robot	207 (82.5%)	63 (80.8%)		158 (90.8%)	134 (91.8%)	

**Table 2.** Demographic comparison between primary and external cohort.

Characteristics	Primary cohort	External cohort	*p* value
n	329	320	
Age	58.36 ± 10.81	56.59 ± 11.25	0.041
BMI	25.24 ± 3.47	25.46 ± 3.39	0.407
Preoperative creatinine	83.0 (70.0, 97.0)	66.8 (55.8, 77.0)	< 0.001
Preoperative eGFR	83.59 (71.44, 98.79)	97.63 (87.92, 106.43)	< 0.001
Post-to-preoperative serum creatinine ratio	1.28 (1.11, 1.51)	1.37 (1.15, 1.62)	0.002
Tumor size	5.0 (3.5, 7.0)	5.0 (3.5, 6.7)	0.529
Hypertension, *n* (%)			0.727
No	194 (59%)	193 (60.3%)	
Yes	135 (41%)	127 (39.7%)	
Diabetes, *n* (%)			0.129
No	274 (83.3%)	280 (87.5%)	
Yes	55 (16.7%)	40 (12.5%)	
Gender, *n* (%)			< 0.001
Female	211 (64.1%)	118 (36.9%)	
Male	118 (35.9%)	202 (63.1%)	
Surgical approach, *n* (%)			< 0.001
Open	59 (17.9%)	28 (8.8%)	
Laparoscopy or Robot	270 (82.1%)	292 (91.2%)	
label, *n* (%)			< 0.001
No upgrading of CKD	251 (76.3%)	174 (54.4%)	
Upgrading of CKD	78 (23.7%)	146 (45.6%)	

**Table 3.** Demographic comparison of the training set and internal validation set.

Characteristics	Training set	Internal validation set	P value
*n*	229	100	
Age	57.49 ± 10.65	60.34 ± 10.95	0.028
BMI	25.18 ± 3.58	25.38 ± 3.22	0.629
Preoperative creatinine	82.9 (69.0, 97.0)	83.4 (73.0, 95.6)	0.583
Preoperative eGFR	84.15 (72.78, 99.76)	82.01 (69.96, 96.30)	0.244
Post-to-preoperative serum creatinine ratio	1.27 (1.09, 1.51)	1.31 (1.15, 1.47)	0.402
Tumor size	5.0 (3.5, 7.0)	5.0 (3.5, 7.0)	0.739
Hypertension, *n* (%)			0.029
No	144 (62.9%)	50 (50%)	
Yes	85 (37.1%)	50 (50%)	
Diabetes, *n* (%)			0.463
No	193 (84.3%)	81 (81%)	
Yes	36 (15.7%)	19 (19%)	
Gender, *n* (%)			0.641
Female	145 (63.3%)	66 (66%)	
Male	84 (36.7%)	34 (34%)	
Surgical approach, *n* (%)			0.739
Open	40 (17.5%)	19 (19%)	
Laparoscopy or Robot	189 (82.5%)	81 (81%)	
label, *n* (%)			0.934
No upgrading of CKD	175 (76.4%)	76 (76%)	
Upgrading of CKD	54 (23.6%)	24 (24%)	

### 3.2. Model performance

Based on our previous research [5], ten prespecified predictors were incorporated into each machine-learning model: age, gender, BMI, hypertension, diabetes, preoperative serum creatinine, preoperative estimated glomerular filtration rate, post-to-preoperative serum creatinine ratio, tumor size, and surgical approach. The same predictor set was used to develop and compare five machine-learning algorithms, including LightGBM, XGBoost, random forest, support vector machine, and logistic regression.

Model performance was assessed on the internal validation set and evaluated in terms of AUC, accuracy, sensitivity, and specificity. The predictive performance of each model in the internal validation set and the external evaluation cohort is summarized in Table 4 and Table 5. Additionally, the receiver operating characteristic (ROC) curves and the corresponding AUC values for each model in the training set, internal validation set and external evaluation cohort are shown in Figures 3A, 3B and 3C, respectively. The five candidate models were first compared in the internal validation set and subsequently evaluated in the multicenter external evaluation cohort. LightGBM achieved the highest numerical AUC in both cohorts, with an AUC of 0.7508 (95% CI: 0.6399–0.8617) and an accuracy of 0.7200 in the internal validation set, and an AUC of 0.7549 (95% CI: 0.7022–0.8076) and an accuracy of 0.6813 in the external evaluation cohort. Pairwise DeLong tests were performed to compare the AUCs of the five machine-learning models. In the internal validation set, LightGBM showed a significantly higher AUC than XGBoost (*p* = 0.0120), RF (*p* = 0.0360), SVM (*p* = 0.0214), and LR (*p* = 0.0067). In the external evaluation cohort, the AUC of LightGBM was significantly higher than those of XGBoost (*p* = 0.0397), SVM (*p* < 0.001), and LR (*p* < 0.001). However, no statistically significant difference was observed between LightGBM and RF in the external evaluation cohort (*p* = 0.1119). Although LightGBM was not statistically superior to RF in the external evaluation cohort, it achieved the highest numerical AUC in both cohorts and maintained competitive probabilistic prediction accuracy. Considering its discrimination, Brier scores, decision-curve performance, and SHAP-based interpretability, LightGBM was identified as the preferred model. The complete pairwise comparisons are presented in Supplementary Tables S2 and S3. Because results from the external cohort contributed to this comparative assessment and model selection, the external cohort is interpreted as an external evaluation cohort rather than as independent confirmation of a LightGBM model finalized a priori.

**Figure 3.**
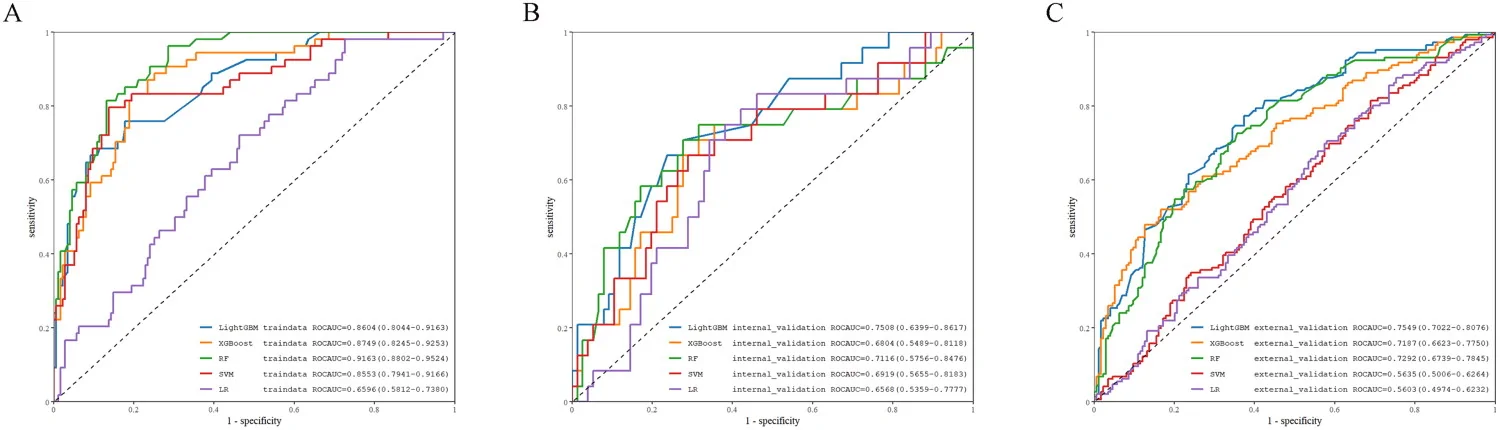
Discriminative performance of machine learning models in the training set(A), internal validation set(B) and external evaluation set(C), evaluated by the area under the ROC curve (AUC). AUC, area under the curve; ROC, receiver operating characteristics. LightGBM, light gradient boosting machine; XGBoost, eXtreme gradient boosting; RF, random forest; SVM, support vector machine; LR, Logistic Regression.

**Table 4.** Comparison of the performance of machine learning models in the training set and internal validation set.

Set	Model	Accuracy	AUC	95% CI	Sensitivity	Specificity	Brier score
Training set							
	LightGBM	0.8079	0.8604	0.8044–0.9163	0.7593	0.8229	0.1288
	XGBoost	0.8122	0.8749	0.8245–0.9253	0.8333	0.8057	0.1267
	RF	0.8559	0.9163	0.8802–0.9524	0.8148	0.8686	0.1126
	SVM	0.8472	0.8553	0.7941–0.9166	0.7963	0.8629	0.1488
	LR	0.5808	0.6596	0.5812–0.7380	0.7222	0.5371	0.1720
Internal validation set							
	LightGBM	0.7200	0.7508	0.6399–0.8617	0.6667	0.7368	0.1558
	XGBoost	0.6900	0.6804	0.5489–0.8118	0.5417	0.7368	0.1697
	RF	0.7000	0.7116	0.5756–0.8476	0.7500	0.6842	0.1595
	SVM	0.6300	0.6919	0.5655–0.8183	0.7083	0.6053	0.1683
	LR	0.6600	0.6568	0.5359–0.7777	0.7083	0.6447	0.1806

AUC: area under the curve; 95% CI: 95% confidence intervals; LightGBM: light gradient boosting machine; XGBoost: eXtreme gradient boosting; RF: random forest; SVM: support vector machine; LR: Logistic Regression.

**Table 5.** Comparison of the performance of machine learning models in the external evaluation set.

Set	Model	Accuracy	AUC	95% CI	Sensitivity	Specificity	Brier score
	LightGBM	0.6813	0.7549	0.7022–0.8076	0.5685	0.7759	0.2553
	XGBoost	0.6656	0.7187	0.6623–0.7750	0.5205	0.7874	0.2519
	RF	0.6719	0.7292	0.6739–0.7845	0.6575	0.6839	0.2566
	SVM	0.5469	0.5635	0.5006–0.6264	0.4521	0.6264	0.2943
	LR	0.5344	0.5603	0.4974–0.6232	0.4863	0.5747	0.3062

AUC: area under the curve; 95% CI: 95% confidence intervals; LightGBM: light gradient boosting machine; XGBoost: eXtreme gradient boosting; RF: random forest; SVM: support vector machine; LR: Logistic Regression.

Calibration curves for the five models in the training, internal validation, and external evaluation cohorts are presented in Figure 4. The Brier score was further used to evaluate overall probabilistic predictive performance. XGBoost achieved the lowest Brier score in the training cohort (0.1267), followed closely by LightGBM (0.1288). In the internal validation cohort, LightGBM achieved the lowest Brier score (0.1558). In the external evaluation cohort, XGBoost had the lowest Brier score (0.2519), while LightGBM showed a comparable value (0.2553). The calibration intercepts and slopes with 95% CIs for the internal and external evaluation cohorts are summarized in Table 6. In the internal validation cohort, LightGBM showed good calibration (intercept −0.29 [95% CI: −1.07 to 0.49]; slope 0.77 [95% CI: 0.16 to 1.38]), with both intervals including their ideal values. In the external evaluation cohort, all models yielded positive calibration intercepts (0.61 for LightGBM [95% CI: 0.27 to 0.95]), indicating systematic underestimation of risk, which is consistent with the substantially higher incidence of CKD progression in the external cohort (45.6% vs. 23.7% in the primary cohort). Calibration slopes below 1 further suggested that the predicted probabilities were more extreme than the observed outcomes, supporting the need for recalibration before application to populations with different baseline risk. The confusion matrices of the five models in the training, internal validation, and external evaluation cohorts are presented in Figures 5–7, respectively.

**Figure 4.**
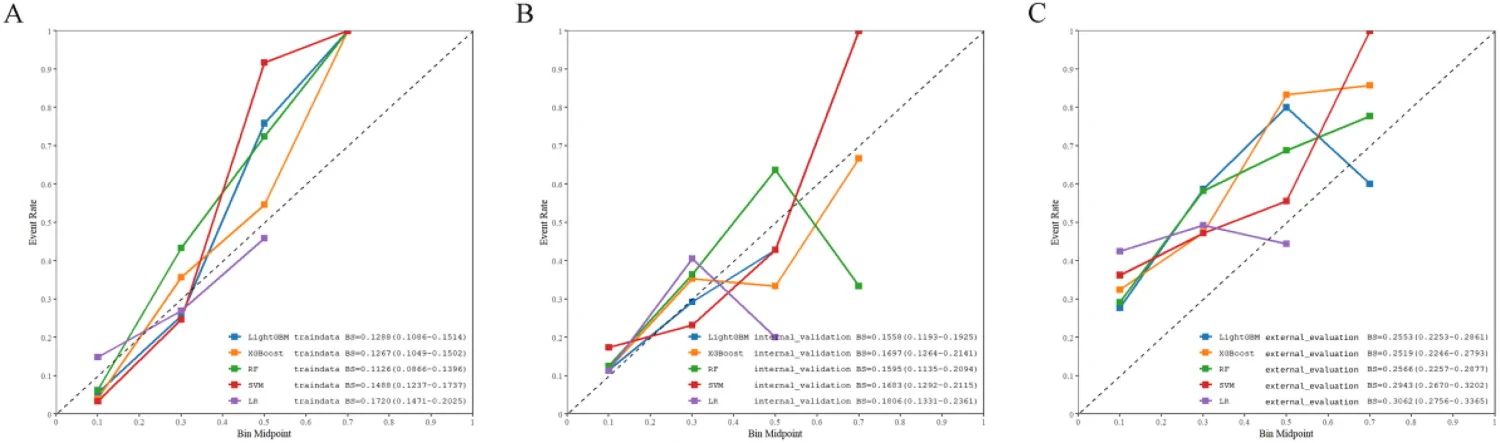
Calibration curves of five machine learning models based on the training set(A), internal validation set (B) and external evaluation set (C).

**Figure 5.**
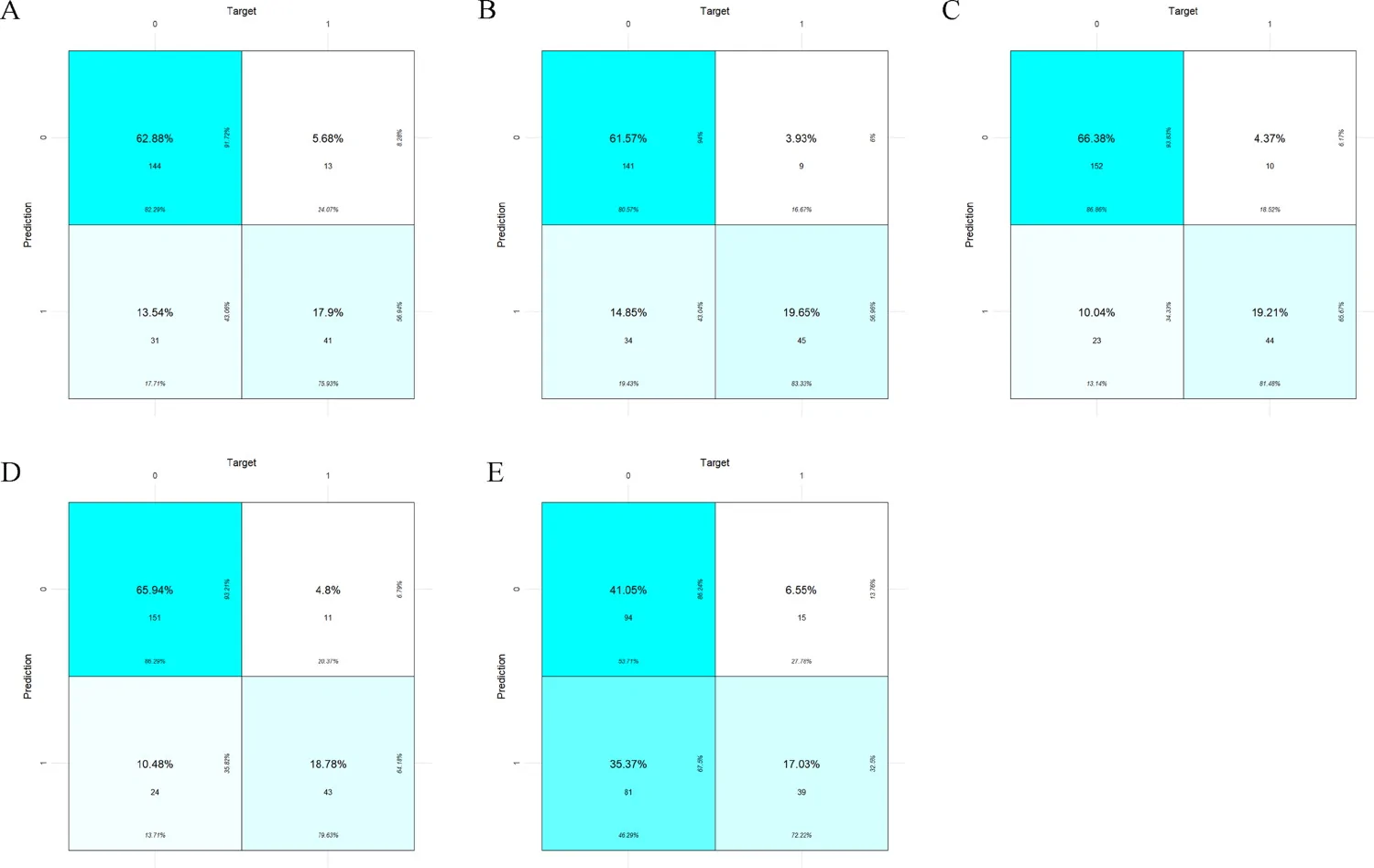
Confusion matrix of the machine learning model based on training set. A: Light Gradient Boosting Machine (LightGBM); B: Extreme Gradient Boosting (XGBoost); C: Random Forest (RF); D: Support Vector Machine (SVM); E: Logistic Regression (LR).

**Figure 6.**
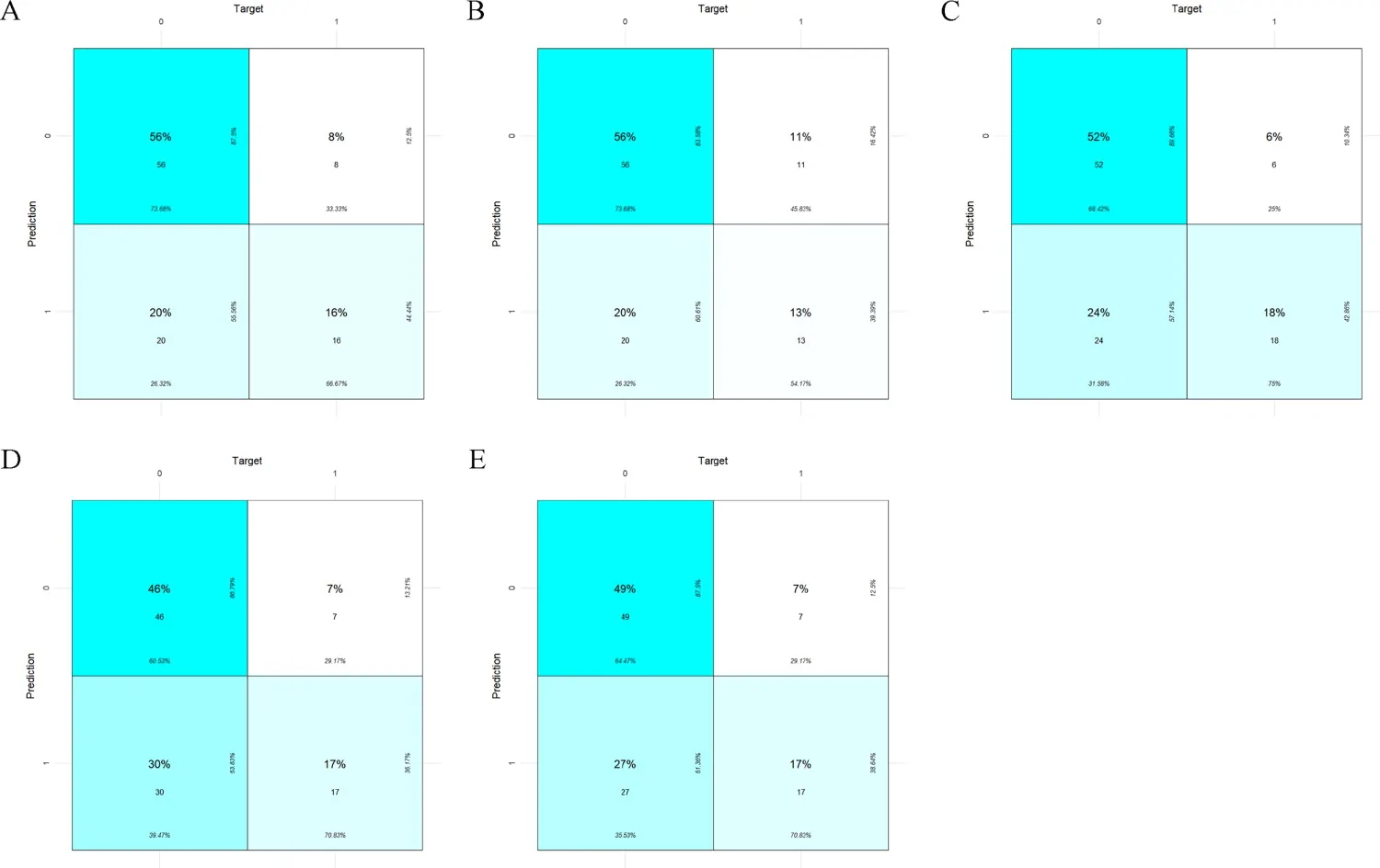
Confusion matrix of the machine learning model based on internal validation set. A: Light Gradient Boosting Machine (LightGBM); B: Extreme Gradient Boosting (XGBoost); C: Random Forest (RF); D: Support Vector Machine (SVM); E: Logistic Regression (LR).

**Figure 7.**
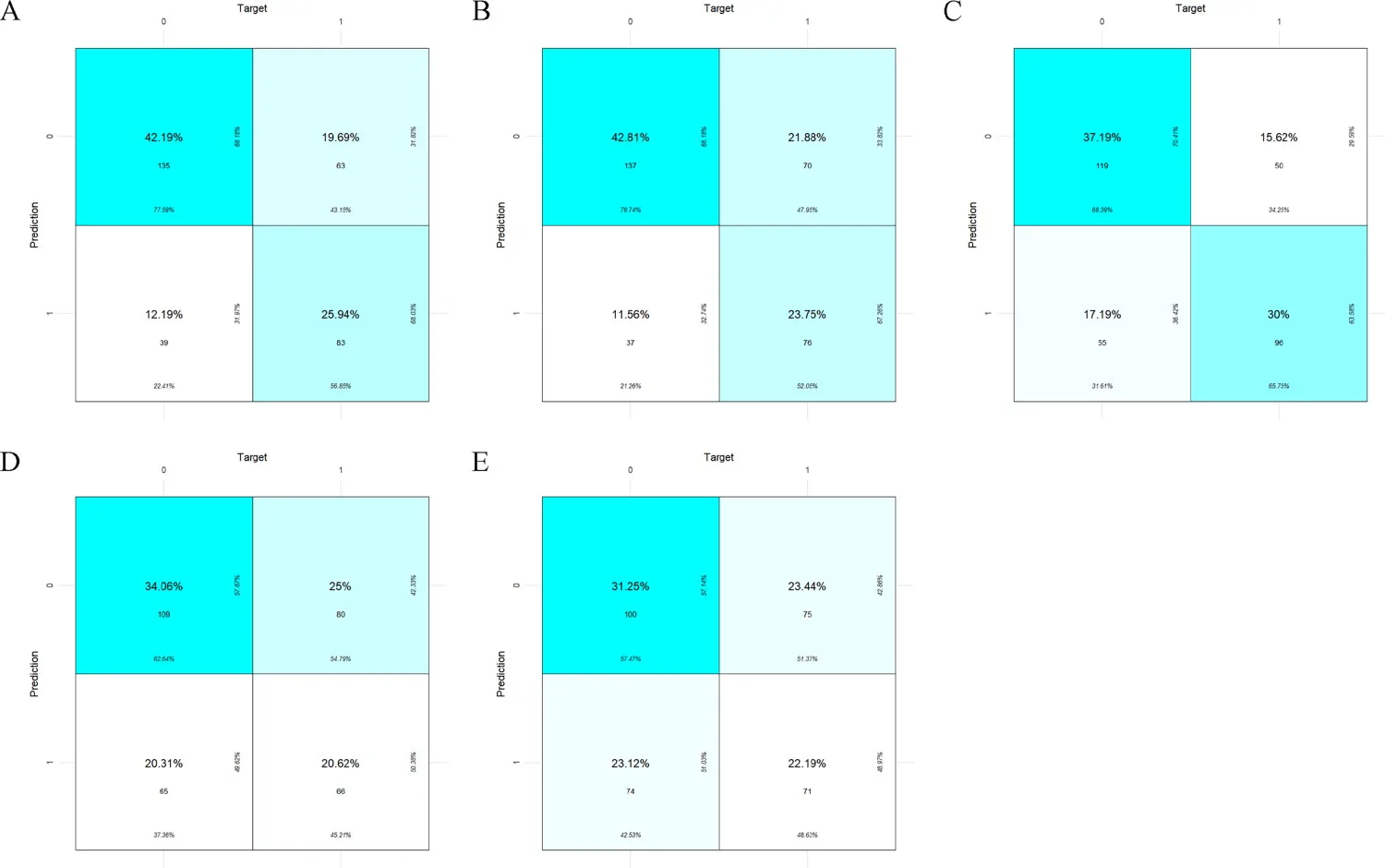
Confusion matrix of the machine learning model based on external evaluation set. A: Light Gradient Boosting Machine (LightGBM); B: Extreme Gradient Boosting (XGBoost); C: Random Forest (RF); D: Support Vector Machine (SVM); E: Logistic Regression (LR).

**Table 6.** Calibration intercept and calibration slope with 95% confidence intervals for the five machine learning models in the internal and external evaluation cohorts.

Cohort	Model	Calibration intercept (95% CI)	Calibration slope (95% CI)
Internal validation cohort			
	LightGBM	−0.29 (−1.07 to 0.49)	0.77 (0.16 to 1.38)
	XGBoost	−0.20 (−0.73 to 0.33)	0.77 (0.33 to 1.21)
	RF	−0.36 (−0.89 to 0.17)	0.53 (0.12 to 0.94)
	SVM	1.10 (0.54 to 1.66)	1.62 (1.14 to 2.10)
	LR	−1.34 (−1.94 to −0.74)	0.09 (−0.34 to 0.52)
External evaluation cohort			
	LightGBM	0.61 (0.27 to 0.95)	0.56 (0.33 to 0.79)
	XGBoost	1.15 (0.78 to 1.52)	0.92 (0.68 to 1.16)
	RF	0.76 (0.41 to 1.11)	0.70 (0.47 to 0.93)
	SVM	1.57 (1.22 to 1.92)	1.29 (1.06 to 1.52)
	LR	−0.19 (−0.50 to 0.12)	0.01 (−0.19 to 0.21)

AUC: area under the curve; 95% CI: 95% confidence intervals; LightGBM: light gradient boosting machine; XGBoost: eXtreme gradient boosting; RF: random forest; SVM: support vector machine; LR: Logistic Regression.

### 3.3. Interpretation of the optimal model with SHAP

To visually elucidate the selected variables, SHAP was utilized to illustrate how these variables predict postoperative CKD stage progression following radical nephrectomy in the LightGBM model. As illustrated in Figure 8, the SHAP summary plot demonstrated that preoperative eGFR was the most influential predictor of CKD stage progression following radical nephrectomy, followed by tumor size, post-to-preoperative serum creatinine ratio, preoperative creatinine et al. This result was similar to our previous research [5]. Regarding the clinical benefit, the DCA curves of the LightGBM model in the internal validation set demonstrated a favorable net clinical benefit across threshold probabilities, suggesting that the model may provide meaningful utility in clinical decision-making (Figure 9).

**Figure 8.**
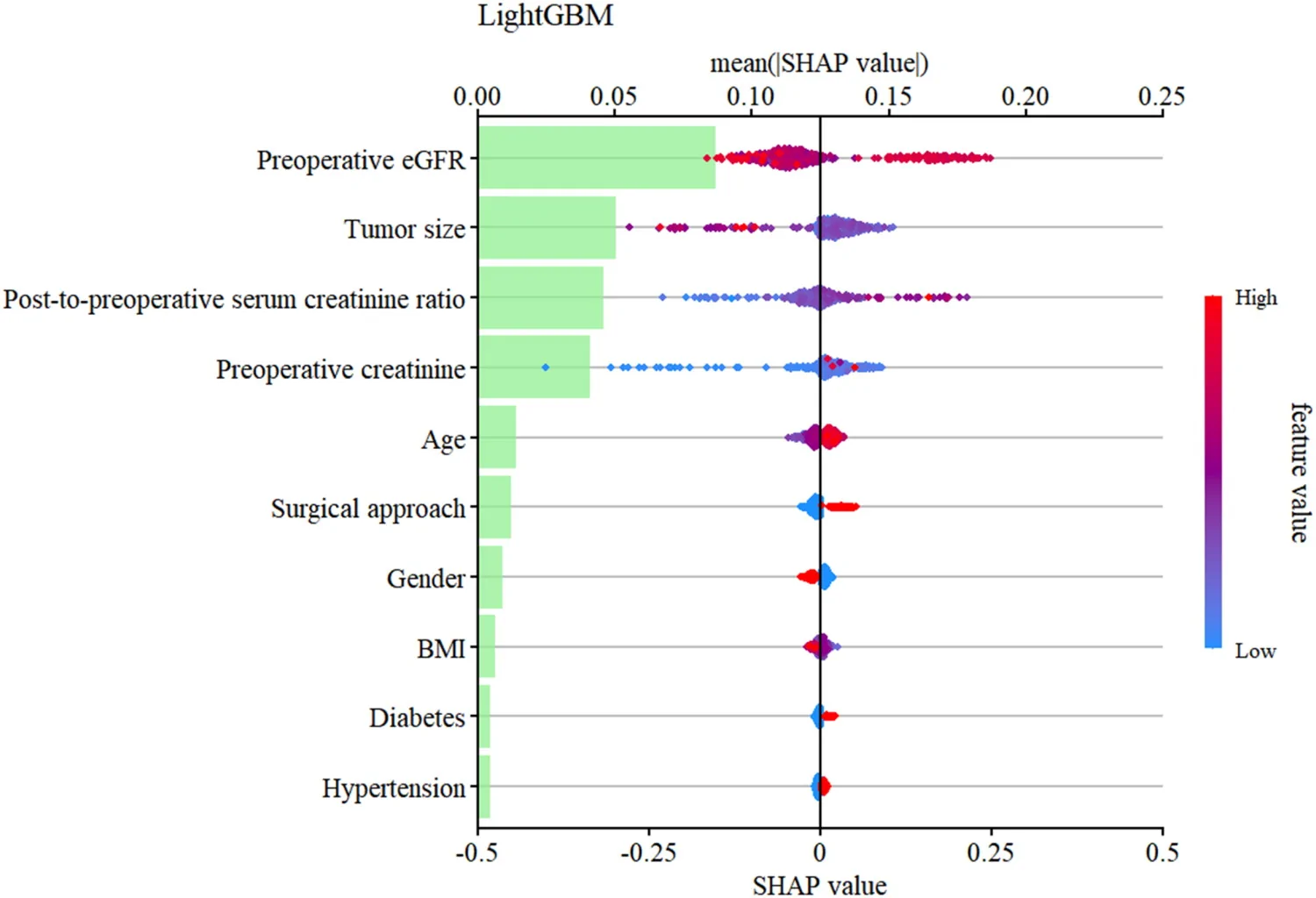
LightGBM Model interpretability based on SHAP analysis.

**Figure 9.**
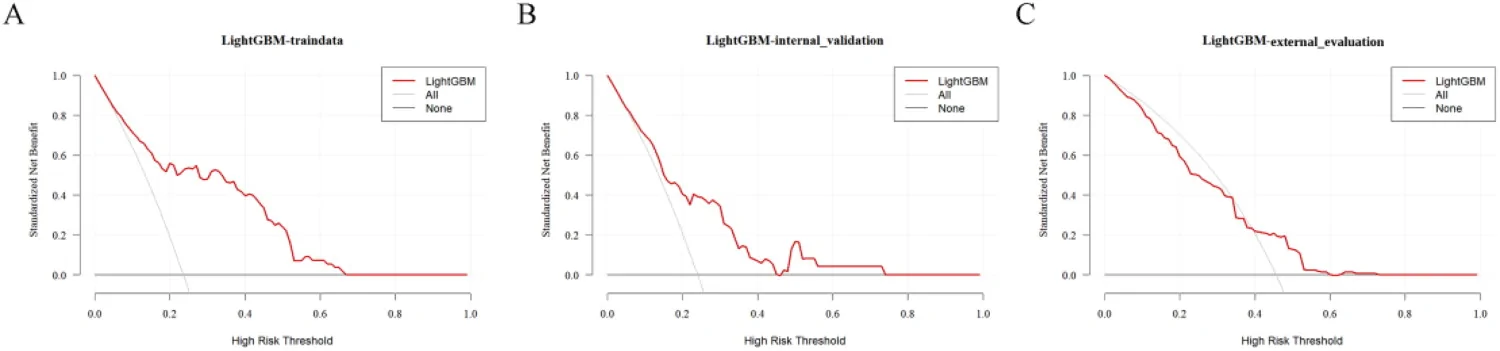
DCA curves of the LightGBM model in the training set(A), internal validation (B) and external evaluation (C).

## 4. Discussion

Radical nephrectomy is the standard treatment for renal cell carcinoma as recommended in guidelines [6]. In the past, partial nephrectomy was only applicable to patients with functional or anatomically solitary kidneys. Therefore, some smaller tumors diagnosed as T1 were also treated with radical nephrectomy. With the gradual development of laparoscopic surgery and the Da Vinci surgical robot, the indications for partial nephrectomy have gradually expanded [7]. Many studies have found that partial nephrectomy can provide better long-term renal function protection compared to radical nephrectomy [8,9]. However, radical nephrectomy is still suitable for larger or deeper renal tumors. Studies have shown that radical nephrectomy can lead to CKD and even dialysis [2,10,11]. The latest EAU guidelines also indicate that long-term follow-up of renal function should be a focus in the follow-up of patients with renal cell carcinoma [12]. This study trained and learned from the data of a single center to establish a predictive model and validated it in multi-center samples, obtaining a reliable predictive model and identifying the relevant influencing factors of long-term changes in renal function after radical nephrectomy for renal cell carcinoma.

In the present study, LightGBM was identified as the preferred model after considering its overall performance across the internal validation and external evaluation analyses, including discrimination, probabilistic prediction accuracy, decision-curve performance, and interpretability. Importantly, because the external cohort contributed to this comparative assessment and the subsequent identification of LightGBM as the preferred algorithm, these external results should not be regarded as fully independent confirmation of a model finalized before external testing. Rather, they provide evidence regarding the comparative performance and transportability of the candidate algorithms across multiple centers. A separate prospective multicenter cohort will therefore be required for fully independent validation of the selected LightGBM model.

Although LightGBM achieved the highest numerical AUC in both validation cohorts, its discriminative ability remained moderate, with AUC values of approximately 0.75. Therefore, the model should not currently be regarded as sufficiently accurate for routine clinical implementation or as a standalone decision-making tool. The favorable net benefit observed in the decision-curve analysis suggests potential clinical usefulness within selected threshold ranges, but DCA alone cannot demonstrate that model-guided care improves patient outcomes. At this stage, the model may be more appropriately considered a preliminary risk-stratification tool that could help identify patients who warrant closer postoperative monitoring. Prospective multicenter validation, assessment of clinical impact, comparison with standard clinical assessment, and recalibration in populations with different baseline characteristics and CKD progression rates are required before routine use.

In the results of this study, among all the relevant parameters included, preoperative renal function (including eGFR and creatinine), tumor size, age, and the ratio of creatinine per- and post-surgery were the most important influencing factors. Preoperative GFR indicated the filtration function of both kidneys. The higher the preoperative GFR, the better the function of both kidneys, and subsequently the better the function of the healthy kidney. On the contrary, many patients may have already had microscopic early pathological lesions in the kidneys due to hypertension, diabetes or other related diseases before surgery [13,14]. Normal creatinine before surgery does not mean normal eGFR [15,16]. Therefore, if the remaining isolated kidney which has been damaged by diseases such as hypertension and diabetes before or after surgery, the risk of CKD progression will increase significantly. The occurrence and development of CKD after surgery is also an important influencing factor for possible cardiovascular events in the future [17]. CKD, cardiovascular events, and systemic metabolic disorders like diabetes, these factors are interrelated and communicate with each other. Studies have shown that cardiovascular events are one of the important causes of death for patients with renal carcinoma [18]. Therefore, for patients who already have CKD before surgery, or those with poorly controlled hypertension or diabetes, the postoperative follow-up of their chronic disease management in the internal medicine is extremely important.

The size of the tumor is a protective factor in our study. However, from a clinical perspective, larger tumors should not be considered beneficial to patient survival. Currently, with the widespread use of health checkup, the early detection of kidney cancer has gradually led to smaller and smaller surgical kidney cancers [19,20], and thus the normal volume occupation and damage within the kidneys have also become smaller. However, larger kidney tumors still occupy the entire volume of the kidney and encroach upon normal renal tissue, ultimately leading to a reduction in functional nephron. Therefore, if the tumor size is larger, the remaining normal nephron are fewer. The bodies of these patients may have adapted to the long process of tumor growth and the reduction of normal nephron over time, so the contralateral kidney also slowly compensates and proliferates due to the destruction of the diseased kidney tissue on the affected side. At the same time, due to the radical nephrectomy, the loss of normal nephron is also relatively small, so the fluctuation of the patient’s own renal function is also lower. On the contrary, if the kidney tumor is smaller, the remaining normal renal tissue on the affected side is also more, and the sudden loss of normal nephron due to the surgery is greater. At this time, the healthy contralateral kidney cannot compensate or proliferate quickly enough, so it is more likely to experience a decline in renal function. Yan et al’.s study found that among patients undergoing different types of kidney resections, the perioperative AKI of the non-functional kidney and the healthy donor kidney were the lowest and highest groups respectively, and they believed that this was related to the sudden loss of effective renal units [4]. Given that radical nephrectomy rarely involves pathological conditions such as ischemia-reperfusion injury that damage the renal parenchyma, we still tend to attribute this finding to the loss of functional nephrons in the affected kidney, the early compensatory hyperplasia of the contralateral kidney during tumor development, and the body’s adaptation to nephron loss. Of course, we must emphasize that this divergence between oncological outcomes and renal function outcomes is not a desirable scenario. Nevertheless, from the perspective of long-term follow-up management, we need to adopt individualized management strategies with different emphases for different patients.

Age has been proved as an extremely important influencing factor for the decline of renal function [21]. Moreover, as age increases, the measurement of creatinine cannot fully reflect the actual functional level of the kidneys [22,23]. Simply calculating creatinine is not sufficient; more emphasis should be placed on the use of the calculated eGFR [24,25]. Focusing only on the level of creatinine after surgery is also one of the clinical blind spots of urologists.

The post-to-preoperative serum creatinine ratio, which may serve as an indicator of both the occurrence and severity of postoperative AKI, is also an important influencing factor for predicting long-term renal function. Perioperative AKI is a common complication of radical nephrectomy, with an incidence rate of over 40% [26–28]. However, other types of AKI (such as ischemia-reperfusion injury, drug-induced injury, etc.) after occurrence will cause long-term chronic damage or fibrosis to the kidney, thereby leading to a decline in renal function [29,30]. For patients with nephrectomy, AKI is mainly a transient decrease in the body’s tolerance capacity [31], while the renal parenchymal cells of the healthy contralateral kidney have not been exposed to any damaging factors. However, studies have shown that after nephrectomy, the blood perfusion of the contralateral kidney will significantly increase, thereby posing a risk of damage to the microscopic structure of the kidneys [32,33]. And AKI is a strong predictor of CKD after nephrectomy [27,34]. However, we have no way to determine the specific pathological changes of the originally healthy contralateral kidney in these patients after the occurrence of perioperative AKI through pathological biopsy. Although many studies have explored the specific biological mechanisms behind renal compensation [35], the pathological changes that this special type of AKI causes to the healthy contralateral kidney are currently unknown. As defined in the KDIGO clinical practice guideline, a postoperative serum creatinine increase to more than 1.5 times the preoperative value defines perioperative AKI, which may herald sustained hyperperfusion and elevated pressure in the healthy contralateral kidney, and obviously this higher pre-renal pressure will cause continuous pathological damage and functional decline of the kidneys. If the pre-renal high pressure of the patient before surgery was low, then the fluctuations during the perioperative period will be small, and AKI will not be manifested, while the other side of the kidney may also reduce the risk of high pressure and the possibility of long-term damage.

Furthermore, we also observed certain discrepancies between the internal and external datasets. Given the current regional imbalance of medical resources in China and the substantially improved transportation accessibility, patients at different stages of the same disease may exhibit distinct preferences in selecting medical centers for treatment, they constitute a stringent test of model transportability. Although discrimination remained stable in the external cohort (AUC 0.7549 vs. 0.7508 internally), stable discrimination alone does not establish model transportability. Consistent with this, the calibration analysis showed that the models were well calibrated in the internal validation cohort—for example, LightGBM achieved a calibration intercept of −0.29 (95% CI: −1.07 to 0.49) and a slope of 0.77 (95% CI: 0.16 to 1.38), with both intervals encompassing their ideal values. In the external cohort, calibration intercepts were positive across models, a pattern consistent with the substantially higher event rate in this population (45.6% vs. 23.7% internally); notably, XGBoost retained a calibration slope close to unity (0.92, 95% CI: 0.68 to 1.16). Such intercept shifts are well recognized when prediction models are transported to populations with different baseline risk and can be readily addressed by routine recalibration, a standard step in model deployment across settings (Table 6).

This study has certain limitations. Firstly, the strict inclusion and exclusion criteria may introduce some selection bias, which may limit the diagnostic applicability of this model in more diverse and real-world populations. Future work should expand the range of patients to ensure its accurate and broad applicability. Secondly, although a multicenter external cohort was included, this cohort was used to evaluate all candidate algorithms, and its results contributed to the identification of LightGBM as the preferred model. Therefore, it does not constitute a fully independent validation of a LightGBM model finalized a priori. The selected LightGBM model should consequently undergo subsequent validation in a separate, independent prospective multicenter cohort before clinical implementation. Thirdly, we did not include CT-based estimation of residual normal renal parenchymal volume of the affected kidney prior to surgery, tumor complexity scores, characteristics of the contralateral kidney, or perioperative complications. The inclusion of these factors may potentially improve our model. Fourthly, the patients included in this study all underwent surgery in accordance with the past indications. However, as mentioned earlier, the indications for radical nephrectomy have been greatly changed, and the patient profile is quite different from before. Therefore, the applicability and parameters of this model may also require more fine-tuning based on future surgical methods or changes in standards. Lastly, the CKD outcome in our study was ascertained on the basis of a single follow-up measurement. In future prospective studies, the stability of patients’ eGFR at follow-up should be assessed, so as to avoid measurement inaccuracies caused by eGFR fluctuations arising from transient patient conditions (such as diet, medication, and other factors).

## 5. Conclusions

We developed and externally evaluated machine-learning models for predicting CKD stage progression within three years after radical nephrectomy. LightGBM was identified as the preferred algorithm after comparative assessment across the internal validation set and external evaluation cohort; because the external cohort contributed to comparative model selection, subsequent independent prospective multicenter validation of the selected model is warranted. The long-term decline in renal function is a very important complication after radical nephrectomy for renal cancer, and it requires the attention and early prevention by urologists during follow-up.

## Supplementary Material

Supplementary Material.docx

## Data Availability

The datasets used and/or analyzed during the current study are available from the corresponding author on reasonable request.
