# Supplementary material for: Identification and validation of a chronic kidney disease prediction model after radical nephrectomy: a multicenter cohort retrospective study
Source: Ann Med. 2026 Sep 29;58(1):2737504. doi: 10.1080/07853890.2026.2737504 (PMC13629762; doi:10.1080/07853890.2026.2737504)
Supplement: Supplementary file 1 [file IANN_A_2737504_SM1673.docx]

**Supplementary Table S1.** Optimal Hyperparameters values for the machine learning models.

| **Model** | **Hyperparameter** | **Optimal Value** |
| --- | --- | --- |
| LightGBM | mtry | 8 |
|  | trees | 466 |
|  | min_n | 9 |
|  | tree_depth | 4 |
|  | learn_rate | 0.074785 |
|  | loss_reduction | 0.77806 |
| XGBoost | mtry | 4 |
|  | min_n | 16 |
|  | tree_depth | 4 |
|  | learn_rate | 0.078421 |
|  | loss_reduction | 0.71828 |
|  | sample_size | 0.83543 |
| RF | mtry | 10 |
|  | trees | 496 |
|  | min_n | 49 |
| SVM | cost | 18.766 |
|  | rbf_sigma | 0.016209 |

LightGBM, light gradient boosting machine; XGBoost, eXtreme gradient boosting; RF, random forest; SVM, Support Vector Machine.

**Supplementary Table S2.** Pairwise comparison of AUCs among machine learning models using DeLong’s test in the internal validation set.

| Model | XGBoost | RF | SVM | LR |
| --- | --- | --- | --- | --- |
| LightGBM | 0.0120 | 0.0360 | 0.0214 | 0.0067 |
| XGBoost |  | 0.0441 | 0.3016 | 0.1861 |
| RF |  |  | 0.2613 | 0.0314 |
| SVM |  |  |  | 0.0418 |

LightGBM, light gradient boosting machine; XGBoost, eXtreme gradient boosting; RF, random forest; SVM, Support Vector Machine; LR, Logistic Regression**.**

**Supplementary Table S3.** Pairwise comparison of AUCs among machine learning models using DeLong’s test in the external validation set.

| Model | XGBoost | RF | SVM | LR |
| --- | --- | --- | --- | --- |
| LightGBM | 0.0397 | 0.1119 | < 0.001 | < 0.001 |
| XGBoost |  | 0.3407 | < 0.001 | < 0.001 |
| RF |  |  | < 0.001 | < 0.001 |
| SVM |  |  |  | 0.0864 |

LightGBM, light gradient boosting machine; XGBoost, eXtreme gradient boosting; RF, random forest; SVM, Support Vector Machine; LR, Logistic Regression**.**
